# Development of a novel clinical prediction model for sepsis related mortality by combining NEWS, PIRO and lactate

**DOI:** 10.17305/bb.2025.12562

**Published:** 2025-06-30

**Authors:** Ozge Kurtkulagi, Ece Unal Cetin, Fatih Kamis, Murat Das, Esen Simsek, Ozgur Kurtkulagi, Adil Ugur Cetin, Yavuz Beyazit

**Affiliations:** 1 Department of Internal Medicine, Faculty of Medicine, Çanakkale Onsekiz Mart University, Çanakkale, Türkiye; 2 Department of Emergency Medicine, Faculty of Medicine, Çanakkale Onsekiz Mart University School of Medicine, Çanakkale, Türkiye; 3 Department of Anesthesiology and Reanimation, Faculty of Medicine, Çanakkale Onsekiz Mart University School of Medicine, Çanakkale, Türkiye; 4 Department of General Surgery, Çanakkale State Hospital, Çanakkale, Türkiye; 5 Department of Internal Medicine, Çanakkale State Hospital, Çanakkale, Türkiye; 6 Department of Gastroenterology, Faculty of Medicine, Çanakkale Onsekiz Mart University, Çanakkale, Türkiye

**Keywords:** Sepsis prognosis, risk assessment, intensive care unit, ICU, blood lactate levels

## Abstract

Prognostic assessment plays a crucial role in guiding therapeutic decision-making for patients with sepsis, particularly in intensive care settings. This study aimed to develop a multivariable model to predict 28-day mortality among intensive care unit (ICU) patients with sepsis by integrating serum lactate levels, the National Early Warning Score (NEWS), and the Predisposition, Infection, Response, and Organ Dysfunction (PIRO) score. Demographic information, clinical characteristics, and laboratory findings routinely collected at ICU admission were used to calculate the NEWS and PIRO scores for each patient. Patients were categorized as survivors or non-survivors based on their outcome. Both logistic regression and Cox proportional hazards models were applied for mortality prediction analysis. The final analysis included 205 patients diagnosed with sepsis (mean age: 73.6 ± 13.2 years; 53.2% male), of whom 109 died during hospitalization. Logistic regression analysis revealed that lactate, NEWS, and PIRO scores were independently associated with 28-day mortality. Combining lactate levels with NEWS and PIRO significantly enhanced mortality prediction, with the greatest accuracy observed when all three parameters were integrated. Pairwise analyses demonstrated that adding lactate to the base model significantly improved predictive accuracy (DBA: −0.103, *P* ═ 0.003), and incorporating lactate into a model already including NEWS further enhanced its predictive value (DBA: −0.042, *P* ═ 0.037). In conclusion, serum lactate measured at initial ICU admission provides valuable prognostic information for predicting 28-day mortality in sepsis patients. Furthermore, combining lactate levels with NEWS and PIRO scores substantially enhances the accuracy of mortality prediction in these patients.

## Introduction

Sepsis—a life-threatening condition resulting from a dysregulated host response to infection—remains a major challenge for intensive care unit (ICU) patients worldwide [1]. Early identification and appropriate management during the initial hours are therefore critical to guiding timely and effective interventions. To aid in this, clinicians often rely on scoring systems, laboratory markers, and predictive models to stratify the risk in sepsis patients [2]. However, there remains a lack of standardized tools that effectively evaluate the prognosis of patients with sepsis after ICU admission. Among the commonly used clinical scoring systems are the National Early Warning Score (NEWS), the Predisposition, Infection, Response, and Organ dysfunction (PIRO) score, and the quick Sequential Organ Failure Assessment (qSOFA) score, all of which have gained popularity for their ability to identify patients at risk of clinical deterioration [3–6]. The NEWS system aggregates vital signs into a single composite score, providing a broad assessment of a patient’s clinical condition [7]. However, it may not fully capture disease severity, as it does not directly incorporate metabolic or inflammatory markers—key components in the pathogenesis of sepsis. The PIRO model was designed as a framework to enhance risk stratification by considering premorbid conditions, infection characteristics, the host’s physiological response, and the degree of organ dysfunction [3, 8, 9]. These four elements collectively encompass many of the factors influencing the onset, progression, and outcome of sepsis. Nonetheless, studies comparing PIRO to other prognostic tools have reported mixed results regarding its predictive accuracy [10–12]. Blood lactate—a well-established biomarker of tissue hypoperfusion and metabolic dysfunction—is frequently elevated in severe sepsis and has been shown to predict both morbidity and mortality [13, 14]. Because lactate levels can be measured quickly and easily, they are widely used in ICUs to assess tissue perfusion in critically ill patients. Elevated lactate is associated with poor outcomes, even in the absence of overt organ dysfunction [13, 15, 16]. Incorporating validated laboratory parameters such as lactate into existing clinical scoring systems like NEWS and PIRO may improve their ability to identify high-risk patients more accurately. These enhanced models could better inform treatment decisions, optimize resource allocation, and ultimately improve patient outcomes. To that end, we developed a multivariable risk stratification model for mortality by integrating serum lactate, the PIRO score, and the NEWS in patients with sepsis. We hypothesized that incorporating lactate into these scoring systems would yield superior predictive accuracy compared to using each predictor alone.

**Table 1:** Baseline characteristics of patients admitted to the ICU, categorized by survival status

**Variables**	**All patients (*n* ═ 205)**	**Survivors (*n* ═ 96)**	**Deceased (*n* ═ 109)**	***P* value**
*Demographic*				
Age (years)	73.6±13.2	71.8±13.1	75.2±13.2	0.064
Sex/Male, *n* (%)	109 (53.2)	51 (46.8)	58 (53.2)	0.990
Intubation (*n*)	88 (42.9)	13 (14.8)	75 (85.2)	<0.001
*ICU admission vital*				
Heart rate (/min)	102.1±22.4	98.9±20.9	104.8±23.4	0.480
Respiratory rate (/min)	22.0±5.1	21.9±5.1	22.1±5.2	0.886
SBP (mmHg)	103.2±23.1	103.3±23.4	103.1±23.0	0.554
MAP (mmHg)	77.2±15.8	76.6±15.0	77.8±16.6	0.431
Temperature (^∘^C)	36.8±0.8	36.7±0.8	36.8±0.7	0.812
*Complete blood count*				
WBC (×10^3^/uL)	14.3±8.8	13.9±8.1	14.6±9.5	0.566
Hemoglobin (g/dL)	10.0±2.1	9.8±2.0	10.3±2.1	0.109
Hematocrit (%)	31.2±6.5	30.6±6.4	31.6±6.6	0.296
Platelet (×10^3^/uL)	234.4±248.8	261.7±323.3	210.3±153.1	0.141
NLR	20.3±21.1	19.4±20.6	21.2±21.5	0.542
MLR	0.83±0.71	0.87±0.76	0.79±0.70	0.459
*Biochemical measurements*				
Blood glucose (mg/dL)	155.9±92.1	148.2±58.0	162.9±113.9	0.238
Urea (mg/dL)	110.6±67.8	94.8±58.9	124.4±72.4	0.002
Creatinine (mg/dL)	2.23±1.68	2.1±1.8	2.3±1.6	0.307
Total bilirubin (mg/dL)	1.3±2.3	1.4±1.9	1.6±2.3	0.773
Sodium (mmol/L)	138.4±8.8	138.5±9.3	138.4±8.4	0.994
Potassium (mmol/L)	4.2±0.9	4.0±0.9	4.4±1.0	0.008
ALT (U/L)	78.7±283.3	46.4±132.3	107.3±366.8	0.108
AST (U/L)	126.9±417.7	56.2±126.1	189.2±554.3	0.016
LDH (U/L)	404.2±495.3	335.9±402.9	464.2±559.3	0.059
Fibrinogen (mg/dL)	492.7±232.4	509.9±220.7	477.6±242.2	0.322
D-dimer (ug/mL)	7.45 ± 9.81	6.6±7.9	8.2±11.2	0.226
CRP (mg/L)	192.1±101.8	185.9±98.6	197.5±104.7	0.419
Sedimentation (mm/h)	55.3±35.5	59.7±36.4	51.4±34.4	0.096
Procalcitonin (ng/mL)	19.8±29.85	17.1±27.7	22.1±31.6	0.227
*Illness acuity assessment tools*				
NEWS	8.4±3.4	7.2±2.9	9.6±3.3	<0.001
PIRO	4.2±1.8	3.6±1.8	4.7±1.7	<0.001
Shock Index	1.04±0.35	1.01±0.31	1.08±0.39	0.166
*Blood gas analysis*				
pH	7.34±0.13	7.38±0.12	7.29±0.14	<0.001
HCO_3_ (mmol/L)	21.2±6.7	22.6±6.4	20.0±6.8	0.006
Lactate (mmol/L)	3.1±3.4	2.1±1.8	3.9±4.1	<0.001
Ionized calcium (mmol/L)	1.1±0.17	1.1±0.17	1.1±0.17	0.764

SBP: Systolic blood pressure; DBP: Diastolic blood pressure; WBC: White blood cell; NLR: Neutrophil lymphocyte ratio; MLR: Monocyte lymphocyte ratio; ALT: Alanine aminotransferase; AST: Aspartate aminotransferase; LDH: Lactate dehydrogenase; CRP: C-Reactive protein; NEWS: National early warning score.

## Materials and methods

### Study design, patient recruitment, and data gathering

The present study was conducted as a retrospective cohort study in the Internal Medicine ICU of Çanakkale Onsekiz Mart University (COMU) Hospital. It was approved by the Clinical Research Ethics Committee of the COMU Faculty of Medicine (Approval No: 2023-154, Date: 18/10/2023). As the study was retrospective and did not involve any identifiable personal data, the requirement for informed consent was waived by the ethics committee. Patient data were retrieved from the hospital’s electronic medical database for the period between June 2021 and December 2023. Data collection included demographic information, comorbidities, functional and physical status, sources of infection, initial venous lactate levels, mechanical ventilation status, and final diagnoses. Additional variables such as mortality-related factors, treatments administered, chronic medical histories, and discharge status were also recorded. Clinical examinations and initial laboratory tests were conducted within 12 h of ICU admission. All patients were followed from ICU admission until hospital discharge or death. Sepsis was defined according to the Third International Consensus Definitions for Sepsis and Septic Shock (Sepsis-3) as an infection accompanied by signs of organ dysfunction, identified by a SOFA score of two or higher [17]. Each patient’s NEWS and PIRO score were also calculated. The NEWS, which ranges from 0 to 20 with higher scores indicating greater severity, evaluates seven physiological parameters: body temperature, heart rate, respiratory rate, supplemental oxygen requirement, blood oxygen saturation, systolic blood pressure, and mental status. The PIRO score was determined according to the original criteria published by Moreno et al., assigning a maximum of eight points across four domains: PIRO. This includes factors such as age, comorbidities, infection source and type, systemic inflammatory response, and the presence of organ failure [3]. Exclusion criteria were: (1) diagnosis of sepsis not meeting Sepsis-3 criteria; (2) pregnancy or breastfeeding; (3) presence of malignant tumors or hematological disorders; (4) missing data for any key variables, including NEWS, PIRO, or lactate; (5) documented ‘do-not-resuscitate’ orders; (6) diagnosis of autoimmune diseases; (7) hepatic or renal insufficiency; and (8) death within the first 24 h of hospital admission. Based on these criteria, 72 patients (25.8%) were excluded, and 205 patients were included in the final analysis.

### Statistical analysis

Descriptive statistics were reported as mean ± standard deviation (SD) for normally distributed variables and as counts with percentages (%) for nominal variables. The Shapiro–Wilk test was used to assess the assumption of normality. Differences in mean values between groups were evaluated using the *t*-test, while categorical variables were compared using Pearson’s chi-square test. Odds ratios (95% CI) for independent clinical parameters were calculated using both univariable and multivariable logistic regression models to predict 28-day mortality. A multivariable logistic regression model was further developed using stepwise variable selection, including variables with a univariable *P* value less than 0.25. The Hosmer–Lemeshow test was applied to evaluate the goodness of fit of the multivariable models, with a nonsignificant *P* value indicating an adequate fit. A covariate-adjusted receiver operating characteristic (ROC) curve, derived from the multivariable logistic regression model, was used to assess diagnostic accuracy and to calculate the area under the ROC curve (AUROC). Pairwise comparisons of AUROCs were performed using the DeLong test. As this was a single-center retrospective study with a limited sample size, the same dataset was used for both model development and evaluation. Alternative validation methods, such as split-sample validation, bootstrapping, and cross-validation, were not employed. All statistical analyses were performed using SPSS version 22.0 for Windows (IBM Corp., Armonk, NY, USA), with a *P* value of less than 0.05 regarded as statistically significant.

## Results

**Figure 1.**
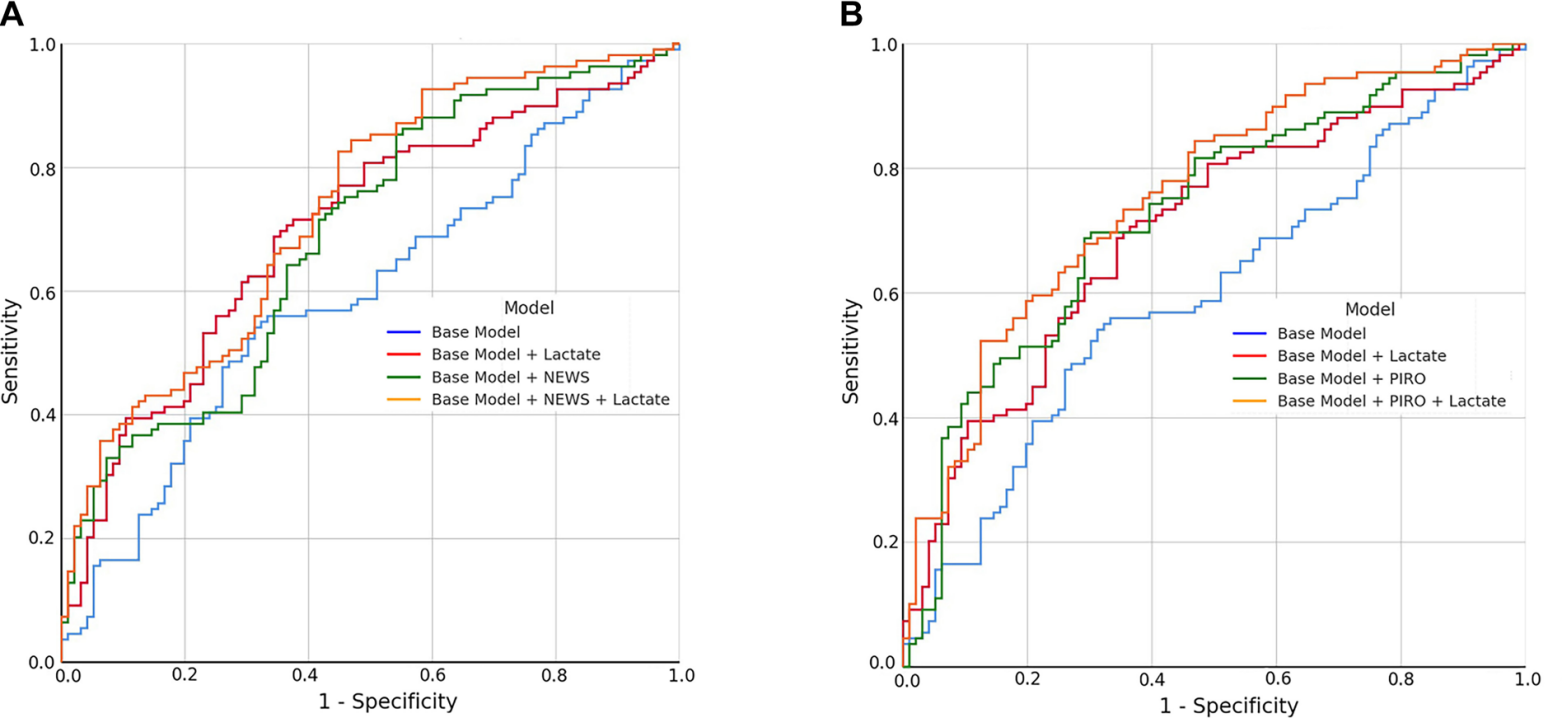
**ROC curves illustrating the predictive performance of various models for 28-day mortality in sepsis patients.** (A) ROC curves for the base model, and models incorporating serum lactate and the National Early Warning Score (NEWS); (B) ROC curves for the base model, and models incorporating serum lactate and the PIRO score. ROC: Receiver operating characteristic.

This study included 205 adult patients with sepsis who met the Sepsis-3.0 clinical criteria. Of these, 109 (53.2%) were male and 96 (46.8%) were female, with a mean age of 73.6 ± 13.2 years. Based on 28-day outcomes, patients were divided into two groups: the survivor group [*n* ═ 96; 51 (53.2%) male, 45 (46.8%) female; mean age 71.8 ± 13.1 years] and the deceased group [*n* ═ 109; 58 (53.2%) male, 51 (46.8%) female; mean age 75.2 ± 13.2 years]. The clinical characteristics and laboratory values of the survivor and deceased groups are shown in Table 1. Deceased patients had significantly higher mean levels of urea, potassium, aspartate aminotransferase (AST), and lactate compared to survivors. The mean NEWS score was also elevated in deceased patients (Table 1). Univariable analysis showed that each 1-point increase in PIRO score, lactate level, and NEWS was associated with an increased mortality rate, with odds ratios of 1.580 (95% CI: 1.277–1.955), 1.284 (95% CI: 1.119–1.473), and 1.234 (95% CI: 1.124–1.355), respectively. Multivariable logistic regression identified PIRO score, NEWS, intubation, urea, pH, and lactate as significant independent predictors of mortality in patients with sepsis (Table 2). Table 3 presents the predictive performance of NEWS, PIRO, and lactate for 28-day mortality in ICU patients with sepsis. Mortality prediction varied significantly across subgroups stratified by NEWS, PIRO score, and lactate level. Patients with a NEWS ≥8 and a PIRO score ≥ 4 had the highest odds ratio: 7.504 (95% CI: 3.189–17.658) in the crude model and 7.240 (95% CI: 2.953–17.752) in the adjusted model. Similarly, patients with a NEWS ≥ 8 and lactate ≥ 2.2 mmol/L had odds ratios of 11.180 (95% CI: 4.500–27.774) in the crude model and 10.779 (95% CI: 4.319–26.902) in the adjusted model (Table 3). We further analyzed the impact of lactate levels on the predictive accuracy of various mortality models (Table 4). A base model was initially developed to identify high-risk patients, incorporating variables such as advanced age, male sex, and elevated procalcitonin levels. Pairwise analysis showed that adding lactate to the base model significantly improved 28-day mortality prediction (DBA: −0.103, *P* ═ 0.003). Similarly, adding lactate to the base model plus NEWS also significantly enhanced predictive accuracy (DBA: −0.042, *P* ═ 0.037). ROC curve analysis was conducted to compare the predictive performance of lactate when combined with the base model, NEWS, and PIRO. Both combinations significantly predicted mortality: base model + lactate (AUROC ═ 0.700, *P* < 0.001) and base model + NEWS + lactate (AUROC ═ 0.732, *P* < 0.001) (Figure 1A). The same analysis was performed for the base model, base model + PIRO (AUROC ═ 0.725, *P* < 0.001), and base model + PIRO + lactate (AUROC ═ 0.756, *P* < 0.001) (Figure 1B).

**Table 2:** Univariable and multivariable logistic regression analyses for predicting mortality

	**Death (*n* ═ 109)**			
	**Univariable analysis**		**Multivariable analysis**	
	**Odds ratio (95% CI)**	***P* value**	**Odds ratio (95% CI)**	***P* value**
Age (years)	1.031 (0.998–1.065)	0.066		
Gender M[F(Ref)]	0.771 (0.350–1.698)	0.990		
NEWS	1.234 (1.124–1.355)	<0.001	1.203 (1.081–1.338)	0.001
PIRO	1.580 (1.277–1.955)	<0.001	1.404 (1.156–1.704)	0.001
Shock Index	1.741 (0.793–3.821)	0.167		
Procalcitonin (ng/mL)	1.006 (0.996–1.015)	0.228		
CRP (mg/L)	1.001 (0.998–1.004)	0.417		
Intubation	14.084 (6.915–28.684)	<0.001	8.853 (3.925–19.965)	<0.001
Urea (mg/dL)	1.007 (1.002–1.012)	0.003	1.006 (1.000–1.012)	0.035
Potassium (mmol/L)	1.494 (1.104–2.021)	0.009		
AST (U/L)	1.002 (1.000–1.004)	0.029		
pH	0.019 (0.002–0.186)	0.001	0.016 (0.001–0.467)	0.016
HCO_3_ (mmol/L)	0.943(0.902–0.985)	0.008		
Lactate (mmol/L)	1.284 (1.119–1.473)	<0.001	1.268 (1.084–1.484)	0.003

NEWS: National Early Warning Score; CRP: C-reactive protein; PIRO: Predisposition, Infection, Response, and Organ Dysfunction; AST: Aspartate aminotransferase; HCO_3_: Bicarbonate.

**Table 3:** Mortality of patients admitted to the intensive care unit for sepsis, stratified by NEWS, PIRO, and serum lactate levels

		**Odds ratio (95% CI)**			
**NEWS | PIRO**	***n* died/*n* total (%)**	**Crude model**	***P***	Adjusted model*	*P*
NEWS<8 | PIRO<4	10/38 (26.3)	Reference		Reference	
NEWS≥8 | PIRO<4	9/29 (31.0)	1.200 (0.433–3.665)	0.671	1.229 (0.419–3.609)	0.707
NEWS<8 | PIRO≥4	23/46 (50)	2.800 (1.110–7.060)	0.029	2.696 (1.016–7.153)	0.046
NEWS≥8 | PIRO≥4	67/92 (74.4)	7.504 (3.189–17.658)	<0.001	7.240 (2.953–17.752)	<0.001
*NEWS/LACTATE*					
NEWS<8 | Lactate<2.2	15/54 (27.8)	Reference		Reference	
NEWS≥8 | Lactate<2.2	33/68 (48.52)	2.451 (1.144–5.253)	0.021	2.428 (1.126–5.238)	0.024
NEWS<8 | Lactate≥2.2	18/30 (60.0)	3.900 (1.520–10.008)	0.005	4.095 (1.577–10.633)	0.005
NEWS≥8 | Lactate≥2.2	43/53 (81.1)	11.180 (4.500–27.774)	<0.001	10.779 (4.319–26.902)	<0.001

*The adjusted model included age and gender. NEWS: National Early Warning Score; PIRO: Predisposition, Infection, Response, and Organ Dysfunction Score.

**Table 4:** Impact of serum lactate on the discriminatory accuracy of mortality prediction models

	**Area under the ROC** **(95% CI)**		**Pairwise analysis**					
	**Without lactate**	**With lactate**			**95% CI**			
**Prognostic model**			**DBA**	**SE**	**Lower**	**Upper**	**Zstatistic**	***P***
Base model ═ Age, Sex, Procalcitonin	0.597 (0.519–0.675)	0.700 (0.629–0.772)	−0.103	0.273	−0.170	−0.036	−0.3023	0.003
NEWS	0.678 (0.605–0.751)	0.727 (0.659–0.796)	−0.049	0.264	−0.092	−0.007	−2.263	0.024
Base model+NEWS	0.690 (0.618–0.762)	0.732 (0.664–0.800)	−0.042	0.263	−0.082	−0.002	−2.083	0.037
PIRO	0.722 (0.653–0.792)	0.757 (0.691–0.823)	−0.035	0.258	−0.082	0.012	−1.461	0.144
Base model+PIRO	0.725 (0.656–0.795)	0.756 (0.690–0.821)	−0.030	0.260	−0.075	0.014	−1.331	0.183

NEWS: National Early Warning Score; PIRO: Predisposition, Infection, Response, and Organ Dysfunction Score.

## Discussion

Baseline clinical findings, laboratory parameters, and metabolic profiles are widely recognized as effective predictors of mortality, particularly in ICU patients. These variables allow clinicians to stratify patients into risk categories, enabling tailored interventions and appropriate levels of care. Utilizing such predictors helps optimize resource allocation, forecast disease progression, and enhance patient outcomes through evidence-based decision-making. Within this context, the present study evaluates the predictive performance of various risk models in estimating clinical outcomes across different scenarios. Our findings indicate that elevated lactate levels, NEWS, and the PIRO score are significantly associated with 28-day mortality, both in crude and adjusted multivariable logistic regression analyses. Models incorporating lactate showed superior predictive accuracy compared to those that did not, underscoring lactate’s critical role in risk assessment. Pairwise comparisons of models—including lactate with either the base model or NEWS, alone or combined—further improved 28-day mortality predictions. These results emphasize the value of integrating validated laboratory parameters into established risk tools such as NEWS and PIRO for improved mortality estimation in ICU patients with sepsis. Although not formally implemented in this study, exploratory analyses using machine learning methods, such as random forest and decision trees, may further enhance predictive accuracy, particularly in larger datasets. Given the complexity of sepsis—characterized by the interplay of pathogen, host response, and multiorgan dysfunction—biomarkers, either alone or combined with prognostic models, offer valuable clinical guidance once validated [18–20]. This study aimed to analyze the combined impact of laboratory and demographic variables to evaluate inflammatory status, nutritional state, and overall clinical condition in septic patients to better assess prognosis. Initial blood lactate levels (≥ 2.2 mmol/L) emerged as an independent predictor of mortality in both crude and adjusted analyses. Supporting this, prior studies by Noparatkailas et al. [21] and Mikkelsen et al. [22] linked lactate levels ≥ 2 mmol/L to increased mortality and risk of septic shock in non-shock septic patients. Similarly, Filho et al. [13] identified a lactate threshold of > 2.5 mmol/L as optimal for predicting 28-day mortality in severe sepsis. These findings reinforce the role of lactate as a reliable biomarker for mortality risk and highlight its utility in clinical decision-making. While lactate is a well-established prognostic marker, one of this study’s primary aims was to assess its impact on scoring systems like PIRO and NEWS. The PIRO system evaluates four critical domains—predisposition, infection, host response, and organ dysfunction—providing a comprehensive staging framework for sepsis [3, 23]. In our analysis, the PIRO score alone was a significant predictor of mortality and remained influential when combined with variables such as age, gender, and procalcitonin. Furthermore, mortality rates varied significantly within NEWS subgroups based on PIRO scores (Table 3). Although no previous study has examined the combined predictive value of NEWS and PIRO scores, Chen and Li [24] compared PIRO with the MEDS and APACHE II scores for predicting organ dysfunction. All three models were independent predictors of 28-day mortality, with similar AUC values (AUC: 0.889 for ICU admission, 0.744 for 28-day mortality). The observed 28-day mortality rate of 53% in our cohort is relatively high, potentially limiting generalizability. This elevated rate may reflect the severity of illness among our study population, which included a large proportion of elderly patients with multiple comorbidities, many of whom required mechanical ventilation and vasopressor support. These characteristics should be considered when applying our findings to other ICU settings. In this study, the NEWS at admission demonstrated comparable accuracy to lactate and PIRO scores in predicting 28-day mortality among ICU patients with sepsis. Furthermore, incorporating lactate into NEWS-based mortality models significantly enhanced predictive accuracy compared to traditional approaches. This improvement may be attributed to the comprehensive structure of the NEWS system, which evaluates seven distinct physiological parameters. The model incorporating PIRO and lactate scores showed a slightly higher AUROC than the model combining NEWS, lactate, and base scores, although this difference was not statistically significant. This modest advantage may reflect the PIRO system’s inclusion of additional clinical variables, such as comorbidities and infection characteristics, which may offer incremental prognostic value in specific patient subgroups. Additionally, our study highlighted the role of NEWS in stratifying mortality risk across varying levels of PIRO and lactate, as shown in Table 3. Both crude and adjusted models indicated that patients with a PIRO score ≥ 4 and lactate ≥ 2.2 mmol/L had an increased risk of mortality, and the inclusion of NEWS significantly improved the model’s predictive accuracy. Patients with a NEWS ≥ 8 and a PIRO score ≥ 4 had a 5.956-fold higher risk of mortality (95% CI: 2.395–14.810) compared to those with a PIRO score < 4. Similarly, those with a NEWS ≥ 8 and lactate ≥ 2.2 had a 4.561-fold increased risk (95% CI: 1.976–10.527) compared to patients with lactate < 2.2. These thresholds may assist clinicians in quickly identifying sepsis patients at higher mortality risk upon ICU admission. Identifying patients with a NEWS ≥ 8, a PIRO score ≥ 4, or lactate ≥ 2.2 could support early risk stratification, guide clinical decision-making, and prompt timely escalation of care, intensive monitoring, and targeted resource allocation. To our knowledge, this is the first study to evaluate the combined effectiveness of NEWS, PIRO, and lactate scores for mortality prediction in sepsis. While de Groot et al. [25] previously assessed the prognostic performance of NEWS and PIRO in sepsis patients admitted to emergency departments, they found that the discriminative ability of each score—PIRO (AUC: 0.78) and NEWS (AUC: 0.72)—was limited when used individually, especially in patients under 70 years of age. These findings underscore that while standalone scores have limitations, combining them with complementary clinical or laboratory markers can significantly enhance predictive performance. This approach supports the use of integrated models over individual scores for more effective sepsis risk stratification. Our focus on combining NEWS and PIRO scores with lactate stems from their practicality and accessibility. Both NEWS and PIRO rely on routine bedside assessments and clinical observations, making them suitable for early ICU risk stratification. In contrast, more complex systems like SOFA or APACHE II, although robust, often require advanced physiological and laboratory data that may not be readily available upon admission [26]. This study also aimed to determine whether pairing a simple clinical score with a widely available biomarker (lactate) could yield a pragmatic model fit for real-world application. Given its simplicity and reliance on routine clinical data, the proposed model could be feasibly integrated into electronic health record (EHR) systems for real-time mortality risk assessment at ICU admission. EHR integration could enable earlier identification of high-risk patients and timely initiation of appropriate management strategies. Alerts based on predefined thresholds—NEWS ≥ 8, PIRO ≥ 4, or lactate ≥ 2.2 mmol/L—could trigger automated notifications for clinical reassessment and escalation of care, facilitating standardized sepsis treatment pathways. This study has several limitations. First, its retrospective design introduces inherent selection bias. Second, although the combination of NEWS, PIRO, and lactate improved predictive accuracy, the models were not externally validated in an independent cohort, limiting their immediate generalizability. Third, the single-center setting and relatively small sample size reduce statistical power and external applicability. Fourth, the absence of detailed treatment-related variables—such as timing of antibiotics, fluid resuscitation volume, or vasopressor use—restricts interpretation, as these factors can substantially influence outcomes in sepsis. Finally, the same dataset was used for both model development and evaluation. Internal validation techniques such as bootstrapping, k-fold cross-validation, or split-sample validation were not employed. This lack of resampling or external validation increases the risk of overfitting. While our findings offer preliminary insights into the value of combining NEWS, PIRO, and lactate scores, further prospective studies and external validations are required to confirm their utility across diverse patient populations.

## Conclusion

In conclusion, this study highlights the value of combining multiple scoring systems with laboratory markers to improve mortality prediction in ICU patients with sepsis. While standalone systems such as NEWS or PIRO offer notable advantages, their predictive performance improves significantly when integrated with validated parameters like the lactate score. This combined approach enables better risk stratification and more efficient allocation of healthcare resources. Although the findings offer valuable preliminary insights, external validation using independent multicenter cohorts is necessary to confirm the model’s robustness. Future research should prioritize prospective, multicenter validation to determine the model’s applicability across diverse patient populations.
